# Current trends in RNA virus detection through metatranscriptome sequencing data

**DOI:** 10.1002/2211-5463.13626

**Published:** 2023-05-20

**Authors:** So Nakagawa, Shoichi Sakaguchi, Atsushi Ogura, Katsuhiko Mineta, Toshinori Endo, Yoshiyuki Suzuki, Takashi Gojobori

**Affiliations:** ^1^ Department of Molecular Life Science Tokai University School of Medicine Kanagawa Japan; ^2^ Division of Genome Sciences, Institute of Medical Sciences Tokai University Kanagawa Japan; ^3^ Division of Interdisciplinary Merging of Health Research, Micro/Nano Technology Center Tokai University Kanagawa Japan; ^4^ Bioinformation and DDBJ Center National Institute of Genetics Mishima Japan; ^5^ Department of Microbiology and Infection Control, Faculty of Medicine Osaka Medical and Pharmaceutical University Japan; ^6^ Graduate School of Bioscience Nagahama Institute of Bioscience and Technology Japan; ^7^ Computational Bioscience Research Center (CBRC) King Abdullah University of Science and Technology (KAUST) Thuwal Saudi Arabia; ^8^ Research Organization for Nano & Life Innovation Waseda University Tokyo Japan; ^9^ Marine Open Innovation Institute (MaOI) Shizuoka Japan; ^10^ Faculty of Information Science and Technology Hokkaido University Sapporo Japan; ^11^ Graduate School of Science Nagoya City University Japan

**Keywords:** endogenous viral elements, metatranscriptome, RNA virome, RNA‐dependent RNA polymerase, SARS‐CoV‐2

## Abstract

With advances in sequencing technology, metatranscriptome sequencing from a variety of environmental and biological sources has revealed the existence of various previously unknown RNA viruses. This review presents recent major RNA virome studies sampled from invertebrate and vertebrate species as well as aquatic environments. In particular, we focus on severe acute respiratory syndrome coronavirus‐2 (SARS‐CoV‐2) and related RNA virus identification through metatranscriptome sequencing analyses. Recently developed bioinformatics software and databases for RNA virus identification are introduced. A relationship between newly identified RNA viruses and endogenous viral elements in host genomes is also discussed.

## Graphical abstract

This review introduces the progress of RNA virus discovery using large‐scale sequencing data. Particularly, severe acute respiratory syndrome coronavirus‐2 (SARS‐CoV‐2) and related RNA virus identification through metatranscriptome sequencing analyses were summarized. We also discussed development of bioinformatics tools to search for RNA virus identification. Future RNA virus discovery studies are also discussed.

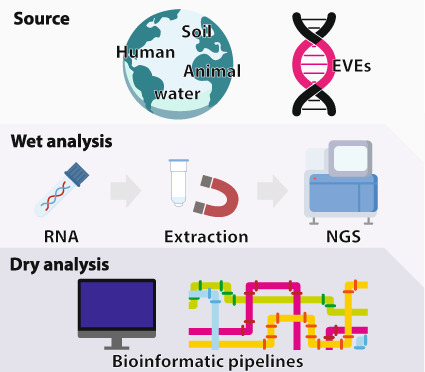

**Abbreviation**
cDNA: complementary DNA
CNN: convolutional neural network
dsRNA: double‐strand RNA
ERV: endogenous retroviruses
EVE: endogenous viral element
FLDS: fragmented and primer ligated dsRNA sequencing
HMM: hidden Markov model
MLP: multilayer perceptron
RdRp: RNA‐dependent RNA polymerase
RNN: recurrent neural network
SARS‐CoV‐2: severe acute respiratory syndrome coronavirus‐2
ssRNA: single‐strand RNA
SVM: support vector machine

Viruses are often classified as something between organisms and material. Although viruses own their genetic materials and successfully reproduce themselves in host cells, they cannot grow and replicate by themselves. Viruses that utilize RNA as a genetic material comprise realms named *Riboviria* and *Ribozyviria* (International Committee on Taxonomy of Viruses, https://talk.ictvonline.org/, access on April 26, 2023). *Orthornavirae* is a major kingdom of *Riboviria* that contains all the double‐strand RNA (dsRNA) and single‐strand RNA (ssRNA) viruses, excluding retroviruses (belonging to kingdom *Pararnavirae*). For their genome replications, RNA viral genomes of *Orthornavirae* code RNA‐dependent RNA polymerase (RdRp), while retroviruses code RNA‐dependent DNA polymerase (i.e., reverse transcriptase). As for *Ribozyviria*, it consists of only a single family *Kolmioviridae* containing *Deltavirus* such as hepatitis delta virus [1]. As RNA genomes of *Ribozyviria* do not code RdRp, they utilize RdRp of other RNA viruses or host replication enzymes for their replications [2]. Furthermore, it is known that some *Eptesicus* bats also code RdRp that was integrated into their genomes [3]. However, despite several exceptions, genes containing the RdRp domain are almost unique to all RNA viruses. Therefore, the presence of RdRp is known as the hallmark of RNA viruses [4]. Recently, many RNA viruses that used RNA as a genetic material were found to exist in various environments, including wild animals [5, 6, 7, 8, 9], oceans [10, 11], and even in the genomes of various species [12, 13, 14, 15, 16, 17, 18]. The discovery of such a large number of viruses in recent years is largely due to advances in DNA sequencing technology. At the same time, the development of bioinformatics technology to analyze such large‐scale nucleotide sequences has made significant contributions. In this review, to understand how metatranscriptome analyses have been used to discover RNA viruses, especially concerning severe acute respiratory syndrome coronavirus‐2 (SARS‐CoV‐2), we first briefly introduce recent major metatranscriptome RNA virome analyses. Then, we summarized bioinformatics tools for RNA virus detection. We also address the endogenous viral elements studies that could be related to novel RNA virus detection.

## Metatranscriptome analysis for the RNA virome

**Fig. 1:**
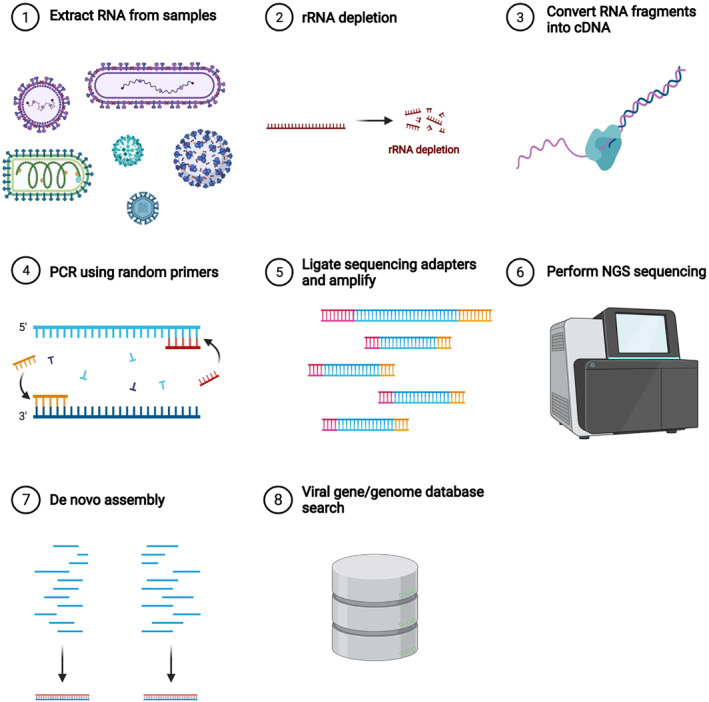
Schematic procedure of a standard metatranscriptome analysis. (1) RNA is extracted from a given sample without enrichment of viruses. (2) Ribosomal RNA (rRNA) is removed. (3) Complementary DNA (cDNA) from the remaining RNA is generated using reverse transcriptase. (4) cDNAs are enriched by PCR using random primers. (5) Adapters are attached for terminals and the cDNAs are amplified. (6) The enriched cDNAs are sequenced. (7) Contigs are constructed by assembling reads. (8) Each contig is searched with viral genome databases. The figure was adapted from ‘RNA sequencing’, by BioRender.com (2022). Retrieved from https://app.biorender.com/biorender‐templates.

RNA viruses have been comprehensively identified using metatranscriptome (RNA metagenome) data. The standard procedure of metatranscriptome analysis is summarized in Fig. 1. First, RNA is extracted from a given sample. After removing ribosomal RNA genes, reverse transcriptase is used to generate complementary DNA (cDNA) from the remaining RNA. Then, cDNAs are enriched by PCR using random primers. The enriched cDNAs are massively sequenced by so‐called next‐generation sequencers. The sequencing data are assembled, and contigs are generated. Note that this assembly step may be unnecessary if long‐read sequencing is applied [19]. Each contig is searched using viral and host genomes and/or genes. Based on the sequence search results, viral genomes are thought to exist in a given sample. Recent advances in sequencing and computer technology have enabled metatranscriptome sequencing at a more affordable price, and many laboratories utilize metatranscriptome methods to identify RNA viruses from various samples.

## Arthropod RNA virome

Since arthropod species transmit various RNA viruses that infect humans, various metatranscriptome analyses of arthropod species have been conducted and revealed a vast diversity of RNA viruses not previously isolated. Li et al. [5] conducted metatranscriptome analysis of 70 arthropod species and identified 112 novel negative‐sense RNA viruses. Shi et al. [6] also conducted a metatranscriptome analysis of invertebrates such as mosquitoes and ticks and reported thousands of previously undetected RNA viruses. Orba et al. [20] sampled mosquitos in the Bolivian Amazon basin and revealed flaviviruses with distinct features. Metatranscriptome analyses of human clinical samples have identified various arthropod‐derived RNA viruses as the cause of the various symptoms. As examples, Alongshan virus belonging to *Jingmenvirus* was found to be associated with human febrile illness [21], while Yezo virus belonging to *Orthonairovirus* was identified in patients showing acute febrile illness with thrombocytopenia and leukopenia after a tick bite [22].

## SARS‐CoV‐2 related RNA virome

One of the most prominent examples of metatranscriptome analyses for RNA virus detection was the discovery of SARS‐CoV‐2 and related coronaviruses. The genome sequence of SARS‐CoV‐2, a coronavirus causing severe respiratory illness named COVID‐19, was revealed by metatranscriptome analyses [23, 24, 25]. Before the COVID‐19 pandemic, the closest coronavirus to SARS‐CoV‐2 was RaTG13, which was isolated from a horseshoe bat (*Rhinolophus affinis*) [26]. In addition, coronaviruses detected in Sunda pangolins (*Manis javanica*) were also found to be similar to SARS‐CoV‐2, particularly with respect to the receptor‐binding domain of the spike protein [27, 28, 29, 30]. Since the start of the pandemic, many metatranscriptome studies have identified various coronaviruses that are closely related to SARS‐CoV‐2 [31, 32, 33, 34]; however, ancestral viruses of SARS‐CoV‐2 have not been found to date (as of April 2023). Furthermore, although a vast metatranscriptome analysis of various wild animals sampled from multiple regions in China revealed a variety of novel RNA viruses, no coronaviruses that were related to SARS‐CoV‐2 were found [8, 9]. Essentially, the evolution of RNA viruses that cause human disease is complex because various RNA viruses that infect humans are not limited to mammals, but closely related RNA viruses also exist in fish [7]. Indeed, a recent study revealed fish could be the first host of the genus influenza virus that also infects humans [35]. Further RNA virome studies will reveal the diversity and evolution of RNA viruses that cause human diseases, including SARS‐CoV‐2.

## Aquatic environment RNA virome

Metatranscriptome analyses have also revealed RNA virus communities in aquatic environments. In one of the oldest studies of the aquatic RNA virome, Culley et al. [36] sampled at Jericho Pier on English Bay and the Strait of Georgia, British Columbia, Canada. A metatranscriptome analysis was conducted with Sanger sequencing technology, and RNA viral contigs belonging to *Picornavirales*, *Tombusviridae*, and *Umbravirus* were obtained. By using 454 sequencing technology which was the first commercially available next‐generation sequencer, Djikeng et al. obtained RNA aquatic samples at Lake Needwood, a freshwater lake in Maryland, USA, and identified various RNA virus‐derived assemblies [37]. Subsequently, various studies have revealed the diversity of RNA viruses in oceans. In a major study, Wolf et al. [38] obtained 101 aquatic samples from Yangshan Deep‐Water Harbor and yielded 4593 nearly full‐length RNA viral RdRps that formed 2192 clusters with 75% amino acid identity. Recently, Zayed et al. [11] analyzed 771 metatranscriptome datasets from 121 locations throughout the world's five oceans and obtained 44 779 contigs, sequences of which 6686 encoded complete or near‐complete RdRp domains. These aquatic metatranscriptome studies indicated the rich diversity of RNA viruses in the world's oceans.

## RNA virome using public databases

Transcriptome data of various species and environments have been stored in public nucleotide sequencing databases, such as SRA [39], ERA [40], and DRA [41]. Kawasaki et al. [42] investigated more than 46 000 public sequencing datasets of mammalian and avian species and identified 882 RNA virus infections of 22 RNA virus families in 695 sequencing datasets from 53 host species. Moreover, Edgar et al. [43] comprehensively analyzed more than 3.8 million datasets of RNA‐seq, metagenome, metatranscriptome, and metavirome stored in the SRA database. 131 957 contigs were found to have < 90% identity with known RdRps that are uniquely found in nonretroviral RNA viruses, suggesting that the analysis expanded the RNA virus diversity about 10‐fold. The viral assembly information is available at their website: https://serratus.io. Neri et al. [44] also analyzed 5150 metatranscriptomes and revealed 124 873 distinct clusters of RNA virus genome segments at 90% identity, including two new candidate phyla and 74 new classes (https://riboviria.org). Those studies clearly suggest that there must be a huge number of RNA viruses that have not been identified to date. Such RNA viruses could also be revealed by metatranscriptome analyses in the future, which will be beneficial for understanding the nature of viruses and their impacts on human health. Meanwhile, no standards exist for the various current RNA virome studies; the quality and definition of RNA viruses reported for each study vary widely. In addition, the definition of RNA taxonomy is frequently revised to accommodate research on large‐scale RNA virus discoveries.

## Development of computational methods

Metatranscriptome analyses to detect RNA viruses largely depend on RNA viral genome sequences stored in databases, as the assembled contigs are usually compared with existing RNA viral genomes to determine whether they are derived from RNA viruses. Owing to the advancement of DNA sequencing technology, the number of RNA viral gene and genome sequences stored in public databases has been increasing. Using such RNA viral gene and genome information, various bioinformatics tools and databases have been developed.

Table 1 summarizes the major software and databases for RNA virus identification available to date (as of April 2022). One of the major approaches utilizes a hidden Markov model (HMM). This is because RNA viral genes are highly divergent even for orthologous genes containing the RdRp domain [45, 46, 47]. An HMM profile based on a multiple sequence alignment can identify distantly related sequences more efficiently than a pairwise‐based search [48]. RNA virus HMM profiles were constructed and distributed by vFams [49], RVDB‐prot [50], efam [51], and VirSorter2 [52]; in particular, RVDB‐prot has been updated frequently and has high potential applicability. NeoRdRp [53], RdRp‐scan [54], and Olendraite et al. [55] also provide HMM‐based databases; however, they target only the RdRp domain as the hallmark of nonretroviral RNA viruses (i.e., *Orthornavirae*). Palmscan with PALMdb also targets RdRp domains utilizing position‐specific scoring matrix approaches [56].

**Table 1:** Bioinformatics tools for RNA virus identification.

Tool	Target	Search method	References	Web
vfams	Virus	HMM	[49]	https://derisilab.ucsf.edu/software/vFam/
rvdb‐prot	Virus	HMM	[50]	https://rvdb‐prot.pasteur.fr/
efam	Virus	HMM	[51]	https://datacommons.cyverse.org/browse/iplant/home/shared/iVirus/Zayed_efam_2020.1
virsorter2	Virus	HMM	[52]	https://github.com/jiarong/VirSorter2
neordrp	RdRp	HMM	[53]	https://github.com/shoichisakaguchi/NeoRdRp
rdrp‐scan	RdRp	HMM	[54]	https://github.com/JustineCharon/RdRp‐scan
(Olendraite et al. 2023)	RdRp	HMM	[55]	https://github.com/ingridole/ViralRdRp_pHMMs
palmscan/ palmdb	RdRp	Position‐specific scoring matrices (PSSMs)	[56]	https://github.com/rcedgar/palmscan
viromatch	Virus	BWA‐MEM, DIAMOND	[63]	https://twylie.github.io/viromatch/
virmine	Virus	BLAST	[64]	https://github.com/putonti/virMine
virusseeker	Virus	BLAST	[65]	https://github.com/guoyanzhao/VirusSeeker‐Virome
virfind	Virus	BLAST	[66]	http://virfind.org/
virkraken	Virus	Kraken2	–	https://github.com/Strong‐Lab/VirKraken
viromescan	Virus	Bowtie2	[67]	https://sourceforge.net/projects/viromescan/
tar‐vir	RNA virus	Bowtie2, BWA	[68]	https://github.com/chjiao/TAR‐VIR
virfinder	Virus	k‐mer based SVM	[69]	https://github.com/jessieren/VirFinder
deepvirfinder	Virus	k‐mer based CNN	[70]	https://github.com/jessieren/DeepVirFinder
cheer	RNA virus	Hierarchically organized CNN	[71]	https://github.com/KennthShang/CHEER
cnn_virus	Virus	Multitask learning model based on CNN	[72]	https://github.com/MaHaoran627/CNN_Virus
rnn‐virseeker	Virus	k‐mer based RNN	[73]	https://github.com/crazyinter/RNN‐VirSeeker
vibrant	Virus	HMM and multilayer perceptron (MLP) neural network	[74]	https://github.com/AnantharamanLab/VIBRANT/
virtifier	Virus	Attention‐based long short‐term memory (LSTM) network	[75]	https://github.com/crazyinter/Seq2Vec

Sequence similarity search programs, such as blast [57, 58], bowtie2 [59], bwa [60], kraken 2 [61], and diamond [62], have been commonly used such as viromatch [63], virmine [64], virusseeker [65], virfind [66], virkraken (https://github.com/Strong‐Lab/VirKraken), viromescan [67], and tar‐vir [68]. Recently developed programs have applied machine‐learning algorithms using *k*‐mer (length of *k* nucleotides) frequencies of viral genes/genomes. virfinder [69] is one of the pioneering virus identification programs that utilizes machine‐learning algorithms. Additionally, other machine‐learning algorithms such as convolutional neural network (CNN; DeepVirFinder [70], CHEER [71], CNN_Virus [72]), recurrent neural network (RNN; RNN‐VirSeeker [73]), and multilayer perceptron (MLP) neural network (VIBRANT [74]), and attention‐based long short‐term memory (LSTM) network (Virtifier [75]) were reported. Since machine‐learning‐based virus identification programs are not based exclusively on sequence similarity to known viruses, they are capable of identifying a wide variety of unknown viruses. Some of those machine‐learning‐based virus identification programs were benchmarked by Glickman et al. [76]. However, with the development of machine‐learning fields such as protein structure prediction and language model, various new algorithmic bioinformatics programs continue to be proposed for virus search as well (please see the ‘Conclusion and future studies’ section as well). Since the purpose of each bioinformatics tool could differ and progress in bioinformatics methods is rapid, it will be necessary to constantly follow the latest papers for appropriate RNA virome analyses.

## Endogenous viral elements and metatranscriptome analyses

Advances in genome research have also revealed many RNA virus‐derived sequences in eukaryotic genomes. For example, approximately 8–10% of the human genome corresponds to retroviral origins [77, 78, 79], called endogenous retroviruses (ERVs). Since retroviruses code an RNA‐dependent DNA polymerase (i.e., reverse transcriptase) that can replicate DNA based on an RNA template, the retroviral RNA genome is transcribed to DNA and can be integrated into the host genome using integrase. By chance, some of these are integrated into germline cells, which then become part of the host genome [80]. Since RNA viruses other than retroviruses do not encode reverse transcriptases, the portion of nonretroviral RNA viruses is quite limited; however, some exist in the host genome, which are commonly called endogenous viral elements (EVEs) [11, 12, 13, 14, 15, 16, 17]. Metatranscriptome analyses have directly or indirectly identified EVEs in host genomes. Edgar et al. [43] estimated that around 1% of RNA viral contigs obtained through their metatranscriptome analyses correspond to EVEs. Furthermore, the recent discovery of many new RNA viruses facilitates the finding of previously unidentified EVEs. Kawasaki et al. employed carbovirus and cultervirus sequences that are newly identified bornavirus species as queries and identified various bornavirus‐derived EVEs in a variety of mammalian species [16]. This observation clearly indicates that there are many unidentified EVEs in host genomes. Indeed, Kojima et al. [81] applied a *k*‐mer‐based support vector machine (SVM) classifier using EVE sequences as a training dataset and identified possible EVEs with low similarity to known RNA viruses in the human genome. Such EVEs could also be informative in the search for novel RNA viruses in nature. On the contrary, Kryukov et al. and Irwin et al. reported various viral contaminations in host genome data [15, 17], which may be incorrectly detected as EVEs. Further analyses and development of sequencing technology may be needed to understand the evolution of EVEs in detail.

## Conclusion and future studies

Metatranscriptome analyses have revealed a variety of RNA viruses in various hosts and environments. However, it has also been shown that the RNA viruses found to date represent only a small subset of all RNA viruses. One of the bottlenecks in RNA virus identification from metatranscriptome data is its reliance on similarity to known RNA virus sequences. As noted, machine‐learning‐based methods could be useful; however, it is also difficult to confirm whether those detected sequences are derived from RNA viruses or not. To overcome such problems, a sequencing method named ‘fragmented and primer ligated dsRNA sequencing’ (FLDS) has been developed [10, 82, 83, 84]. Since long dsRNAs are rare in eukaryotic cells, they are mainly derived from the genomes of dsRNA viruses or replication intermediates of ssRNA viruses [85]. Utilizing the biochemical properties of RNA viruses, sequences obtained by FLDS are likely to be RNA viruses even if they are not necessarily highly similar to known RNA viruses. Moreover, FLDS determines entire dsRNA sequences, including both terminals, and therefore, multisegmented genomes of RNA viruses can be reconstructed based on their terminal sequence similarities [82]. In addition to the FLDS methods, the three‐dimensional structure of proteins is known to be more conserved than the underlying amino acid sequence [86]. Since AlphaFold2 can predict the protein structure of a given amino acid sequence with practical quality [87], RNA viral genes could also be predicted by their estimated three‐dimensional structures. Therefore, more RNA viruses could be identified from metatranscriptome data combining various technologies in the near future.

## Conflict of interest

The authors declare no conflict of interest.

## Author contributions

SN, AO, KM, TE, YS, and TG designed the concept of the manuscript. SN wrote the manuscript. SN and SS made a table. SS made a figure. All authors edited and approved the manuscript.

## Data Availability

Not applicable.
